# Prevention Is the Best Treatment: The Case for Understanding the Transition from Monoclonal Gammopathy of Undetermined Significance to Myeloma

**DOI:** 10.3390/ijms19113621

**Published:** 2018-11-16

**Authors:** Michael H. Tomasson, Mahmoud Ali, Vanessa De Oliveira, Qian Xiao, Yogesh Jethava, Fenghuang Zhan, Adam M. Fitzsimmons, Melissa L. Bates

**Affiliations:** 1 Department of Internal Medicine, Hematology, Oncology, and Bone Marrow Transplant Division, University of Iowa, Iowa City, IA 52242, USA; michael-tomasson@uiowa.edu (M.H.T.); Mahmoud-ali@uiowa.edu (M.A.); vanessa-deoliveira@uiowa.edu (V.D.O.); yogesh-jethava@uiowa.edu (Y.J.); Fenghuang-Zhan@uiowa.edu (F.Z.); 2 Holden Comprehensive Cancer Center, University of Iowa, Iowa City, IA 52242, USA; 3 Graduate Program in Molecular Medicine, University of Iowa, Iowa City, IA 52242, USA; adam-fitzsimmons@uiowa.edu; 4 Department of Health Human Physiology, University of Iowa, Iowa City, IA 52242, USA; qian-xiao@uiowa.edu; 5 Stead Family Department of Pediatrics, University of Iowa, Iowa, IA 52242, USA

**Keywords:** multiple myeloma, prevention, monoclonal gammopathy of undetermined significance, plasma cell disease, therapy

## Abstract

Multiple myeloma is an invariably fatal cancer of plasma cells. Despite tremendous advances in treatment, this malignancy remains incurable in most individuals. We postulate that strategies aimed at prevention have the potential to be more effective in preventing myeloma-related death than additional pharmaceutical strategies aimed at treating advanced disease. Here, we present a rationale for the development of prevention therapy and highlight potential target areas of study.

Multiple Myeloma (MM) is a neoplasm of clonal plasma cells that is characterized by the presence of elevated serum monoclonal protein and end organ damage, collectively known as CRAB features (hyperCalcemia, Renal insufficiency, Anemia, and Bone lesions) [1]. Despite tremendous advances in patient care, MM remains incurable in the majority of patients [2]. The five-year overall survival rate is ~50%, with worse prognosis in older adults and some improvements with the use of newer, novel agents (bortezomib, thalidomide, or lenalidomide) [3,4]. The number of patients with MM is expected to continue increasing because of the increased size of the aging population and overall improvements in medical care that contribute to increased longevity [5].

We offer this review because, like prostate and some breast cancers, MM has a requisite pre-malignant stage that may predate the transition to malignancy by many years [6]. Recent updates to the diagnostic criteria for MM using sensitive magnetic resonance imaging of small bone lesions has pushed diagnosis earlier in the disease process. Developing treatment and prevention strategies in MM is particularly attractive because MM meets 8 of 10 Wilson and Junger criteria for disease screening and prevention (Figure 1). These factors include the long, requisite pre-malignant phase (monoclonal gammopathy of uncertain significance, MGUS), and the significant morbidity and inevitable mortality that accompany a diagnosis of myeloma. Unique to myeloma are the existence of two screening blood tests for MGUS and myeloma that approach 99% sensitivity and specificity, e.g., serum protein electrophoresis and serum-free light chains. There currently are no interventions known to halt the progression of MGUS to MM. In solid tumor cancers, tremendous focus has been placed on preventing the transition from pre-malignancy to malignancy (examples in [7,8,9]). We contend that there are opportunities to apply a similar focus to MM, thereby developing treatments to prevent MM. Indeed, given the resistance of MM to current treatment regimens, the most effective way to cure MM may be to prevent it from developing.

## 1. Multiple Myeloma Is a Terminal Disease that Proceeds Through a Requisite Pre-Malignant Stage

MM is consistently preceded by a pre-malignant condition termed Monoclonal Gammopathy of Undetermined Significance (MGUS). The pre-malignant condition is termed to be of “undetermined significance” because some, but not all, patients will progress to malignancy. The pre-malignant MGUS is rare in the young (0.34% of 10–49 year olds) [10], but more common in those over 50 years old (1–3%) [11]. The incidence of MGUS increases with age and is defined by the presence of serum monoclonal protein of <3g/dL or bone marrow plasmacytosis of <10% in the absence of MM-associated end-organ damage or amyloidosis. MGUS has an annual risk of 1% for progression into MM [12]. This progression occurs with or without an intermediate stage referred to as smoldering multiple myeloma (SMM).

**Figure 2:**
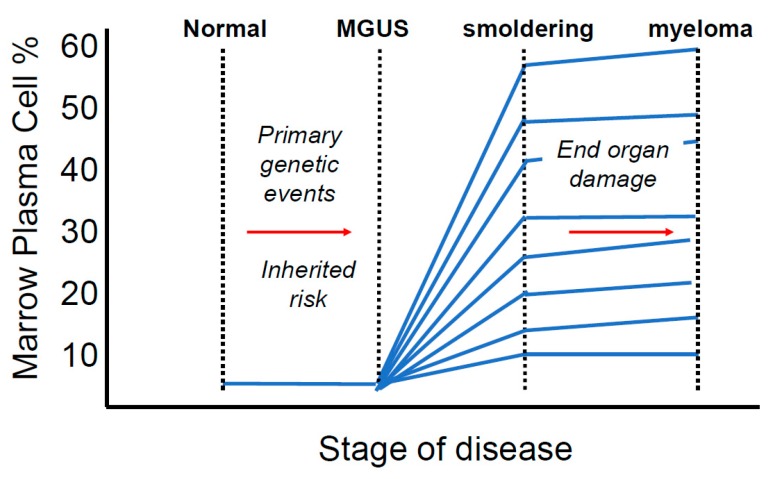
Overview of the development of multiple myeloma. Myeloma progresses through two pre-malignant stages, monoclonal gammopathy of undetermined significance and smoldering myeloma. Although all cases of MM are preceded by MGUS with or without intervening SMM, not all MGUS or SMM cases progress into MM. Therefore, the standard care for MGUS and SMM is careful observation until the development symptomatic MM [14]. Many risk stratification techniques have been suggested to monitor patients with MGUS and SMM and predict those at higher risk of progression for preventive treatment. However, due to the incurable nature of the disease, we and others [6] emphasize the importance of identifying and treating patients at high risk of progression into MM before the onset of symptoms and development of end-organ damage.

SMM is a clinically significant stage between MGUS and MM that has a higher rate of progression into MM, at 10% per year. SMM is characterized by a level of serum monoclonal protein that is higher than that of MGUS at >3 g/dL or bone marrow plasmacytosis between 10% and 60% in the absence of end-organ damage or amyloidosis [13]. Critical to the transition from SMM to MM is the fact that the transition may not necessarily be associated with an increase in bone marrow plasma cells. Rather, end organ damage is the defining feature of MM (see Figure 2). Both MGUS and SMM are frequently asymptomatic conditions and diagnosis is typically made when high monoclonal protein is incidentally discovered on laboratory testing during work up for other disorders. The discovery of MGUS in patients is often not intentional and the initial finding is frequently not made by a hematologist or oncologist.

## 2. MGUS Itself Is Not a Benign Condition

Although pre-malignant condition has been termed to be of “undetermined significance” in terms of the transition to MM, it does not mean that it is not clinically important. The monoclonal antibody population may have characteristics that lead to deposit in or target host tissues. MGUS associated pathologies include sensorimotor neuropathy [15], renal Falconi syndrome [16] and glomerulonephritis [17,18], POEMS [19], capillary leak syndrome [20], ocular disease [21,22], and crystal storing histiocytosis [23,24,25], among others. These may be referred to as “monoclonal gammopathy with clinical significance”. There may be value in defining intervention strategies to reduce monoclonal antibody populations, beyond preventing the MGUS to MM transition.

## 3. Factors Associated with the MGUS to MM Transition

**Figure 3:**
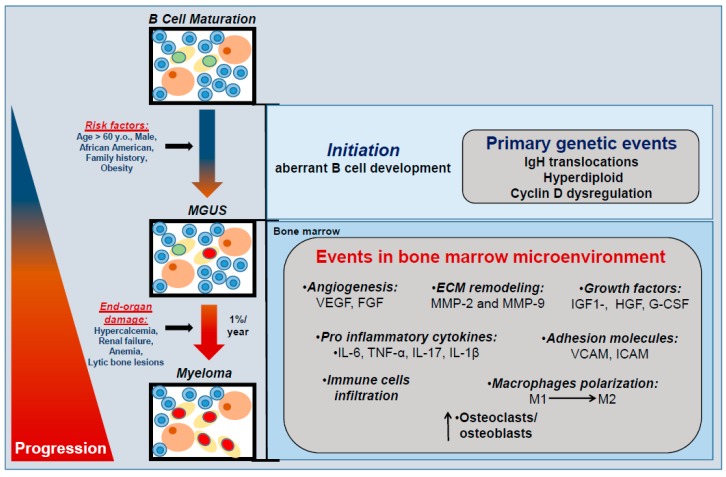
Anthropometric and genetic factors in myeloma initiation and progression. The malignant transformation of a normal B cell to MGUS and then to myeloma is multifactorial. Initiation involves IgH translocations, hyperdiploid, and cyclin D dysregulation. MGUS progression to myeloma is characterized by end-organ damage and remodeling of the bone marrow microenvironment. VEGF, vascular endothelium growth factor; FGF, fibroblast growth factor; MMP-2, matrix metalloproteinase-2; MMP-9, matrix metalloproteinase-9; IGF-1, insulin-like growth factor 1; HGF, hepatocyte growth factor; G-CSF, granulocyte colony stimulating factor; IL-6, interleukin 6; IL-17, interleukin 17; IL-1 β, interleukin 1 β, TNF- α, tumoral necrosis factor-alpha; VCAM, Vascular cell adhesion protein; ICAM, Intercellular adhesion molecule; M1, macrophages M1; M2, macrophages M2.

Compared with other solid tumor cancers that have been subject to multiple, large screening and prevention trials, there have yet been no efforts to try and prevent the development of multiple myeloma. There are several well-identified anthropometric and cytogenetic risk factors for the development of MM, namely age, sex, body mass index, African ancestry, and high risk chromosomal translocation. These, coupled with the key phenotypic changes that occur in the bone marrow that occur in the MGUS to MM transition provide rationale for potential prevention interventions (see summary in Figure 3). We do not presume to offer a comprehensive review of the mechanisms that drive MM, but offer an overview of some topics that may be relevant to prevention researchers.

### 3.1. Race as a Risk Factor in MM

Because MM is the most common hematological malignancy in African Americans [26], the issue of racial disparity in the development of MM is of particular importance in any future prevention strategy. There is likely a strong genetic component to increased risk as it translates not only to Africa Americans, but to increased risk in individuals of Afro-Caribbean and African descent as well [27,28,29]. That said, the molecular and genetic drivers of this increased risk are poorly understood. One hypothesis is that the increased risk of MM in the African American population may from an increased risk of MGUS initiation and that the mechanism of the MGUS to MM transition is the same as compared to white and Hispanic patients. African Americans progress from MGUS to MM at the same rate as their counterparts [30,31], but have an increased overall risk of MGUS. Landgren, et al. studied 12,482 individuals in the National Health and Nutrition Examination Survey (NHANES) III database and found that the incidence of MGUS was nearly twice as high in black participants, compared to white or Hispanic participants [32]. This suggests that the overall increased risk is the result of increased risk of acquiring driver mutations. How or where these genetic lesions occur is unclear. Several human leukocyte antigen alleles have been identified that modify the risk of MM [33,34,35] and there are unique high risk alleles that are identifiable in white, African American and Hispanic populations [35]. However, Baker at al performed tumor sequencing and cytogenetic analysis in African American and European American patients with MM. They found that there was no significance difference in the number or type of somatic copy number aberrations or the incidence of high risk disease, based on genetic sequencing [26]. It is possible the factors that contribute to increased risk are not restricted to variants that impact the B-cell lineage, but may also include genes involved in stromal and immune interactions with the mutated plasma cell that promote its survival and longevity. To date, there have been no comparative DNA or RNASeq studies of the stroma of African American and white MGUS or MM patients. Furthermore, despite evidence that patients of African descent progress to MM at the same rate as other patients, it would be premature to conclude that the mechanism underlying their progression are the same as other populations.

Beyond increased risk of developing MGUS and MM, there are also important race-based differences in care, including decreased access to novel agent therapy and hematopoetic stem cell transplant among African American patients receiving Medicare or Medicaid benefits. [36,37]. Eliminating treatment barriers eliminates race-based outcome disparities [38]. Any study that evaluates the utility of a prevention study must include a racially diverse population because it is possible that (1) there may still be mechanistic differences by which patients of different racial backgrounds progress to malignancy and (2) there may be significant socioeconomic barriers that effect treatment efficacy in different populations.

### 3.2. Prognostic Value of Cytogenetics and Role of Secondary Mutations in Driving the MGUS to MM Transition

Primary and secondary cytogenetic events occur in the progression from MGUS to MM [39]. Primary cytogenetic events occur at MGUS initiation and include trisomies and chromosomal translocations involving the immunoglobulin heavy chain locus of chromosome 14 [40]. Secondary cytogenetic events occur at both the MGUS to SMM transition and during MM progression and include del(17p), del(13q), amp(1q), del(1p), and *Myc* translocation (del indications deletion and amp indicates amplification) [41,42,43,44]. Rajkumar et al. genetically characterized 351 SMM patients at the Mayo Clinic and found that particular phenotypes are associated with shorter time to progression to MM [43]. In particular, the shortest time to progression was found in patients with t(4;14) and del17p. Trisomies, t(11;14) and other immunoglobulin translocations convey intermediate risk and patients with no identifiable abnormalities are at low risk of progression.

Despite epidemiological data that suggests that particular cytogenetic profiles are at increased risk of progression, experimental evidence from our own lab and others demonstrates that genetics alone are not sufficient in mouse models to drive malignancy [45,46,47] The failure of validation of genetic drivers in animal models in MM and the striking genetic diversity in even early MM cancers has impeded progress in understanding the multi-step process by which plasma B cells are transformed into malignant MM cells that invade the bone marrow and destroy cortical bone. Successful mouse models of other hematological malignancies, such as acute myeloid leukemia, have been developed by expressing cancer-associated mutations in the bone marrow, but MM-associated mutations are insufficient to induce MM in mice.

The most common single nucleotide variants (i.e., point mutation) associated with MM in humans are activating mutations in *K-Ras* and *N-Ras* oncogenes [48,49]. We developed several strains of mice expressing an activated *KRas* allele (KRasG12D) in germinal center cells, the precursor to plasma cells and the presumed cell of origin of MM. Using different germinal center Cre recombinase strains (C-gamma-1-Cre and AID-Cre), we found that *KRas* activation was insufficient to induce MM in mice [50]. Remarkably, even after crossing these mice into different tumor prone genetic backgrounds (Ink4a-/-) [45], we found germinal center proliferation, but no trafficking of plasma cells to the bone marrow. In fact, these mice die of an invasive epithelial carcinoma as a result of tiny amounts of off-target Cre recombinase expression, but not MM. The Vk*Myc mouse develops a smoldering phenotype with increased serum immunoglobulin, decreased bone density and monoclonal plasma cells, but only late in life [46], suggesting that aging may promote the development of the disease. Despite extensive efforts, patient-associated mutations have largely failed to faithfully produce disease in an array of mouse models. Together, these data suggest that heritable and somatic mutations alone are not sufficient to explain the progression from MGUS to MM.

### 3.3. Uniquely Dysregulated Genes in Plasma Cells of MGUS

Using gene expression profiles (GEPs), we analyzed differentially expressed genes in the plasma cells of 22 healthy subjects (NPC), 44 MGUS and 351 symptomatic MM patients. A total of 52 genes were identified uniquely dysregulated in MGUS compared to normal donors and MM samples [51]. The majority of dysregulated genes (41 of 52) exhibited a progressive expression increase along the transition from NPC to MGUS to MM. The major functional categories included cell cycling, DNA synthesis, and chromosomal assembly. One gene, *CD27*/*TNFRSF7*, encoded a cell surface and soluble protein, that was progressively down-regulated in the transition from NPC to MGUS to MM to MMCL (MM cell lines). Further study demonstrated that low CD27 expression was associated with a poor prognosis in MM patients [52]. Single cell sequencing studies are underway, which will better delineate deregulated signaling pathways in the MGUS to MM transition. Together, MGUS gene expression signatures may provide valuable molecular targets for the prevention of the multistep molecular pathogenesis of MM.

### 3.4. Antigenic Stimulation

The lipid processing disorder Gaucher’s disease is associated with MM. Chronic exposure to antigenic lipids gives rise to monoclonal gammopathy in Gaucher’s disease reactive to lyso-glucosylceremide [53]. Also, 23% of paraproteins in myeloma and MGUS may be specific for common infectious pathogens (e.g., Epstein Barr Virus, Herpes Simplex Virus, Varicella Zoster Virus, and Helicobater pylori) [54]. These findings suggest that chronic stimulation of B cell activation may play a role of myeloma development in a subset of patients. Reduction of antigenic stimulation or signaling pathways downstream may be a prevention strategy in Gaucher’s and perhaps other patients.

### 3.5. Obesity and Inflammation

We previously reviewed the literature linking obesity with the progression of multiple myeloma [55] and much attention has been given to understanding the mechanism that link these two pathologies. One of the largest prospective studies to evaluate obesity and cancer risk clearly demonstrates an increased risk of myeloma death (relative risk = 1.44) in men and women in with a body mass index of 30–34.9 kg/m^2^ [54]. Obesity is additionally linked with an increased risk of MGUS. In a cross-sectional study of women aged 40–79 years, obese women were significantly more likely to have MGUS (relative risk = 1.8) [56]. Studies evaluating the impact of obesity duration suggest that early life obesity may further increase overall MM risk compared to later life obesity alone [57,58,59].

The mechanism linking obesity and MM is not entirely clear, but may include overlapping contributions of inflammatory and adiposity. In support of a direct role of obesity in promoting MM, reduced expression of the lipokine adiponectin, as occurs in association with obesity, is associated with progression of MGUS to MM in humans [60]. Adiponectin activates protein kinase A, leading to the activation of 5′ adenosine monophosphate- activated protein kinase (AMPK) [61]. Physiologically, AMPK activation inhibits proliferation of MM cells by arresting the cell cycle and promoting apoptosis [61,62]. Through this pathway, decreased adiponectin may lead to increased cell cycling and decreased apoptosis, promoting the survival and proliferation of malignant cells. However, a high fat diet alone, which also decreases adiponectin and enhances inflammation, enhances bone loss but is insufficient to recapitulate a terminal MM phenotype in mice [63]. An additional list of growth factors and cytokines that may be important in myelomagenesis is reviewed here [55].

An “inflammatory-” and/or “angiogenic switch” is also a novel characteristic of the MM bone marrow, in comparison to MGUS. Indeed, the key factors driving progression may be changes in the bone marrow microenvironment and not simply genetic mutations in the B cell lineage. Myeloma-associated macrophages are abundant in MM bone marrow and have been explored as a potentially therapeutic target [64]. The presence of M2-type macrophages in the bone marrow is associated with reduced survival. The macrophages promote MM cell proliferation and survival and may drive angiogenesis via IL-6, TNF-α, and VEGF signaling [65]. Macrophages may communicate with mesenchymal stromal cells and, via cross-talk between the malignant plasma cells, macrophages and stroma, may contribute to the formation of the “myeloma niche” [66]. Targeting these interactions may provide a strategy to prevent the MM transition [67]. In further support of a mechanistic contribute of inflammation to MM progression, modifying IL-6 signaling contributes to disease progression in mouse models. Rutsch et al. developed a double transgenic model with IL-6 and *Myc* transgenes and found that this inflammatory cytokine and oncogene cooperated to induce plasmacytomas within 3–6 months [68]. This model has been used to evaluate chemotherapy efficacy [69]. Constitutive activation of the IL-6 signal transducer GP130 cooperates with *Myc* to cause a MM-like disease in mice with lytic bone lesions, bone marrow plasma cells, and monoclonal gammopathy [70]. Anti-GP130 antibodies prevent tumor formation [71]. Taken together, this suggests that inflammation is a key component of MM and strategies that target inflammation may have utility in preventing the MGUS to MM transition.

### 3.6. Immune Dysregulation

Patients with myeloma are at high risk for infections due to lowered levels of protective antibody titers, but cellular immunity may also be disrupted in MM. Immunosuppressive regulator T cells (Tregs) are elevated mouse models of MM and in smoldering myeloma compared with controls [72]. Murine and patient myeloma cells secrete interferon gamma, which stimulates the proliferation of Tregs contributing to expansion of disease in mice, and possibly immune suppression in patients. The role of Tregs in early stage disease remains unclear, but these results support additional studies on the role of the immune system in the progression of MGUS to MM.

## 4. Potential Therapeutic Targets and Strategies in Preventing Myeloma

### 4.1. Use of Mathematical Models in Studying Prevention

Implementation of a successful screening strategy is a complex undertaking, not without its own costs and risks. The fact that myeloma is a relatively rare cancer (2.1% of all cancer deaths in the US) makes screening more challenging, and screening efforts in other cancers have shown these endeavors to be rife with pitfalls. We developed a population model of disease progression to address the question of whether a myeloma screening and prevention strategy could be beneficial even theoretically, and tested determinants that impacted clinical outcomes [73]. The strength of a mathematical model approach is that it can be used to inexpensively test different screening strategies, and their effects on disease outcome and overall mortality, prior to the design and final implementation of an intervention trial.

We used available epidemiology data on the incidence of MGUS and myeloma and the rates of progression and death to create both mathematical (formula) and agent-based (computational) models. Next, we tested the effects on disease incidence and mortality of screening for MGUS and the implementation of (a hypothetical) effective intervention. We tested the models under various conditions, including the age at first test, the interval between testing, and the relative effectiveness of the intervention (i.e., from 0 completely stops disease progression to 1 has no effect on disease progression). Using the model, we could observe the effect of lead time bias, one of the expected effects of screening. Not surprisingly, screening for MGUS and intervention to stop progression resulted in an increase in the incidence of MGUS and a decrease in the number of myeloma cases. Interestingly, while changing the screening parameters impacted the disease incidence, the myeloma mortality rate was reduced significantly only in high risk groups or with a reduction progression risk reduction. Therefore, we concluded that screening individuals with relatively high lifetime risk of myeloma could provide significant benefits, and improved interventions are needed before screening could provide benefits to groups at a low risk of developing myeloma.

The scientific problem is that the mechanics of disease progression are unclear. A significant barrier to the design of clinical trials in myeloma prevention is our poor understanding of myeloma disease initiation and the early stages of myeloma development are complex. Sequencing has revealed clonal heterogeneity, even at the MGUS stage. Again, model systems may shed light here. Measurement of M proteins in myeloma provides a sensitive and quantitative measure of disease burden, and we used careful analysis of M-protein responses to induction chemotherapy to develop a cellular math model of response dynamics [74]. We analyzed several potential models to explain disease responses in patients and found that a model of a single or multiple equivalent clones in competition could not explain responses. Instead the data supported a hierarchical differentiation model where myeloma cells are sensitive to treatment and myeloma progenitor cells are relatively resistant to chemotherapy. These data reveal functional heterogeneity of myeloma cell clones at presentation.

Furthermore, myeloma initiation may not even involve a single cell type. We sequenced the genome of the myeloma-prone KaLwRij mouse strain to identify genetic pathways that could contribute to myeloma initiation [45]. Surprisingly, the mutations we found in this strain, including a deletion of the *Samsn*1 (*Hacs1*) gene, are not restricted to B-cells, but also affect macrophages and other bone marrow populations to support myeloma development. Therefore, even at its earliest stages, before disease development, in a simple model system, multiple bone marrow cell types are affected. Additional studies of the tumor-stroma microenvironment are warranted to unravel the mechanics of how myeloma begins.

There is a catch 22 in performing a prevention clinical trial because, without broader screening of the underlying asymptomatic condition, we do not know enough about the underlying epidemiology of MGUS and smoldering myeloma. Also, there are health care system barriers to implementation. If we diagnose MGUS and smoldering myeloma widely, we do not have the personnel in place to effectively manage these patients for the next stages of diagnosis and possible intervention. Still, the explosion of diagnostic technology in medicine, including inexpensive, high throughput DNA testing, and patient-initiated diagnostics (i.e., personal cardiac monitoring and DNA screening) has shifted the frame of the screening debate from, “should we test?” to “what should we do with all the data?” How do we collect the data systematically and give wider access to investigators interested in prevention questions [75]?

### 4.2. Obesity Intervention

It is not clear whether weight loss strategies, either through diet and exercise or bariatric surgery, decrease the risk of progression from MGUS to MM. That said, weight loss is already recommended for the prevention and treatment of cardiometabolic disease in obese individuals [59,76,77] and recommending weight loss to obese patients is generally not controversial [78]. However, there are substantial barriers to weight loss that have been well-studied by investigators in the cardiometabolic health sphere. A substantial number of patients do not achieve significant weight loss [79] and bariatric surgery is not without risk and expense [80]. Still, it is unclear whether MGUS patients would be more motivated to participate in a weight intervention program. Engaging scientists in the health promotion field will be important in answering these questions [81,82,83,84] and may provide an additional opportunity to evaluate the impact on MGUS to MM transition.

### 4.3. Metformin

Given its non-specificity, the mechanism by which metformin impacts myeloma is not entirely clear. It may work via its anti-inflammatory function, reducing blood insulin, by targeting AMPK, or other mechanisms. Chang et al. retrospectively studied 3287 MGUS patients in the US Veterans Administration database and found that metformin use of four years or longer was associated with a decreased risk of MM progression (hazard ratio = 0.47, CI = 0.25–0.87) [85]. Reduced MGUS to MM progression in patients using metformin was confirmed in the UK THIN cohort [86]. In cell line models of MM, metformin works synergistically with dexamethasone [87], bortezomib [88], and ritonavir [89] to limit MM cell proliferation. Ectopic expression of the histone-lysine N-methyltransferase NSD2 expression is seen in patients with the t(4;14) translocation [90]. In prostate cancer, metformin cooperates with NSD2 (previously termed *MMSET*) to reduce migration and invasion [91]. To date, there have been no randomized, controlled trials of metformin in MGUS. Metformin may be an attractive prevention therapy, given its well-established safety profile [92] and low cost ($4.00 for 60 tablets at the time of this review).

### 4.4. Vitamin Deficiency

Plasma levels of Vitamin D are reduced in patients with MM [93,94,95], although this is not routinely monitored by most physicians [94]. Vitamin D deficiency does not appear to be associated with worsened survival or high-risk cytogenetic profiles [96]. While there are calls to evaluate Vitamin D status in MM patients [97], it is not clear whether it is a driver of disease or a biomarker. In MGUS patients, Vitamin D supplementation improves markers of bone health and metabolism [98]. Given the low risk profile of Vitamin D supplemental, and the general recommendation, the Vitamin D deficient patients take a supplement, this is potential treatment option [98].

### 4.5. Sleep Disturbance and Intermittent Hypoxia

Despite extensive research in solid tumor cancers, little is known about the role that sleep disturbance plays in the progression of hematological malignancies, including multiple myeloma. Furthermore, several factors related to sleep quality may impact cancer development, including via sleep duration, sleep fragmentation, circadian disruptions, and breathing disturbances that result in intermittent hypoxia. We review the literature here because it is possible that sleep is an additionally modifiable risk factor in preventing the MGUS to myeloma transition. Xiao et al. evaluated sleep quality in 3995 patients from the NHANES cohort and found that obesity is associated with snoring, poor sleep quality, and short sleep duration [99]. This relationship appears to be independent of socioeconomic status and lifestyle factors [100]. Given that sleep quality is highly linked to body weight and metabolic health [101], and that obesity is linked to MM risk, a prospective trial aimed at improving sleep health may reduce obesity, thereby targeting myeloma risk in MGUS patients. Alternatively, sleep duration may impact MM risk independent of obesity. In one of the only studies to directly link sleep quality with MM risk, Gu et al. found an increased risk of MM (Hazard ratio = 2.06, CI = 1.20–3.51) in individuals sleeping < 5 h/night compared to those sleeping 7–8 h/night [102].

Obesity is also tightly associated with sleep apnea. Additional risk factors are highly overlapping with those of MM and include age, male sex, and African American race. Sleep apnea impacts approximately 15% of the population [103] and the pathophysiological effects are driven by the chronic intermittent hypoxia that occurs each time the patient stops breathing. In severe cases, patients stop breathing more than 60 times per hour [104]. Investigators from the Wisconsin Sleep Cohort performed polysomnography in 1522 individuals and followed them prospectively for more than 20 years. Cancer mortality risk exhibited a dose-response type relationship with the severity of sleep disordered breathing [105,106,107] and cancer mortality was highest in individuals with the most severe apnea phenotypes. While these investigators were able to parse out mortality risk for several solid tumor cancers, the study was not sufficiently powered to evaluate blood cancer risk. However, several investigations have reinforced the link between sleep apnea and solid tumor cancers, including melanoma, kidney, and lung cancers (examples in [108,109,110,111]. While the link between sleep apnea and solid tumors is well-established, little is known about the impact of sleep apnea on blood cancer development. At the time of writing of this review, a PubMed search of the terms “intermittent hypoxia” and “myeloma” yielded zero results. A search of the terms “sleep apnea” and myeloma” yielded eight results, none of which investigate a mechanistic link between the two disordered. A single investigation found evidence of vascular remodeling in the bone marrow and increased circulating monocytes [112]. Children with obstructive sleep apnea have higher circulating levels of IgA [113]. Although these studies were not performed in the context of MM, they suggest that intermittent hypoxia has a physiological impact on the bone marrow and the B cell lineage.

**Figure 4:**
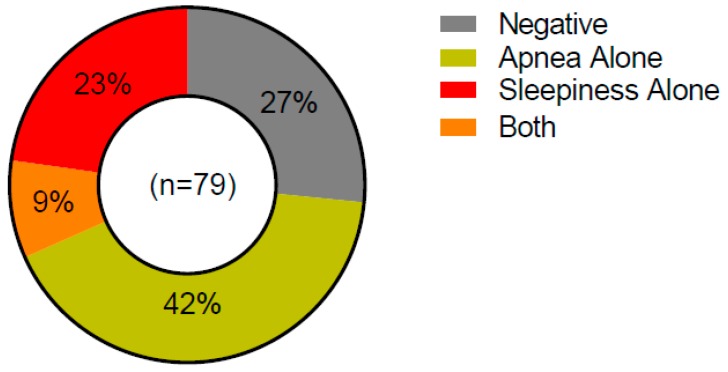
Results of sleep quality questionnaire screening in myeloma patients at the University of Iowa. Patients are identified as at risk of sleep apnea via the Berlin Questionnaire and general sleep disturbance and sleepiness via the Epworth Sleepiness Scale.

We recently began screening MGUS, SMM and MM patients that are seen at the University of Iowa Hospitals and Clinics for sleep disorder using the Berlin Questionnaire [114] and Epworth Sleepiness Scale [115] (University of Iowa IRB #201302833, February 2018) (Figure 4). Interestingly, we identified 51% of patients to be at risk for sleep apnea via the Berlin questionnaire. Forty-two percent of patients were identified as sleepy, via the Epworth Sleepiness Scale. We appreciate that these questionnaires are not a replacement for diagnostic polysomnography, but these data suggest that there is likely undiagnosed sleep disturbance in myeloma patients. Given the strong link between sleep and other malignancies, this is an area that warrants further exploration.

### 4.6. Additional Modifiers of the Inflammatory Response

The prophylactic use of aspirin is often recommended in patients at risk of cardiovascular events [116,117,118]. Birmann et al. evaluated participants in the Nurse’s Health Study and found that adults taking ≥ 5 aspirin (325 mg) per week had a 39% lower MM risk than non-aspirin users [119]. In contrast, meta-analysis of 332,660 found no beneficial effect of regular dose aspirin [120]. To date, there have been no prospective trials of aspirin therapy, but several recent studies have questioned the safety of prophylactic aspirin in aging populations. In a prospective trial of 19,114 elderly patients, daily aspirin use was associated with increased mortality, including increased cancer mortality [121]. Aspirin use is also associated with increased risk of major hemorrhage [122]. There may be important impacts of aspirin dose and body size that contribute to aspirin’s risk-benefit profile [123] and the optimal therapeutic strategy in MM is not clear.

Considering the important role IL-6 has been shown to play in animal models, there may be a role for anti-IL-6 monoclonal antibody therapy for patients at high risk of transitioning from MGUS to MM. The anti-IL6 antibody siltuximab has been evaluated in MM patients and the addition of siltuximab to other treatment regimens does not appear to dramatically improve outcome [124,125]. It is not known whether patients with pre-malignant disease would respond better than patients with fulminant malignancy or if there is a role for monoclonal antibody therapy in MGUS. IL-6 antibody therapy is currently approved for rheumatoid arthritis [126,127]. Given the incidence of rheumatoid arthritis in the population, and overlap of patients with both arthritis and MGUS, it may be possible to mechanistically evaluate the impact of IL-6 antibody therapy in a patient population for whom treatment is already indicated [128,129].

## 5. If Treatments Are Improving, Why Study Prevention in Myeloma?

Myeloma treatments developed over the last 10 years are impressively effective and have improved overall survival significantly. But, with rapid therapeutic advances have come extremely high financial costs. Myeloma drugs are a case study in the skyrocketing costs of cancer drugs. In addition to rising drug costs, MM treatment necessitates weeks spent away from home to receive chemotherapy and/or hematopoetic stem cell transplantation, routine clinic visits, and loss of income associated with disability. Even high-income individuals and those with good insurance find years of deductibles, out of pocket drug costs, and co-pays to cause significant “financial toxicity.” Even successful myeloma treatment is associated with toxicities and morbidity including bone fractures, neuropathy, and pain. Goodwin, et al. surveyed 1015 MM patients and found that 29% lost medical coverage during the course of their treatment [130]. At the time of diagnosis, 66% of patients were employed, compared to only 33% at the time of the survey, including many patients that had not reached retirement age. Participants reported spending 38% of their income on MM-related expenses and one participant reported in a narrative response: *“Medicine was high—Sometimes you have to choose between medicine or food.”*

An accurate accounting and modeling of myeloma treatment costs is a complicated endeavor because drug treatments alone can be broken down into induction regimens, transplantation, maintenance regimen, and relapse regimens, and other healthcare costs related to supportive care, infusion, inpatient care, and emergency care. Stem cell transplantation is thought to be the most expensive part of the treatment of a myeloma patient, but that is not the case. Most recent published data for the maintenance therapy period indicate costs between $22,527 and $47,417 per patient per month (data from 2006–2013) [131]. Contrary to the notion that drug costs are the major driver of cost, anti-MM pharmacy costs contributed to only a quarter of these total costs. At the University of Iowa Holden Comprehensive Cancer Center, the cost of a transplant is about $80,000. We frequently perform tandem transplants, which therefore, therefore cost $160,000 (S. Ouverson, personal communication). We give two years of maintenance chemotherapy, and total cost estimates from the most recent fiscal year indicate that patient care in this two-year period averages $85,000 per patient per month (G. Tricot, personal communication). If we consider that the most recent costs for lenalidomide, bortezomib, and dexamethasone average $18,700 per month [132], then our current costs are comparable to data from 2006–2013, with pharmacy costs contributing to 22% of total patient per month cost. Non-transplant care given for five years at an estimated cost of $125,000 per patient per month could total $7,500,000. Relapse therapy, which has a likelihood of >80% at 10 years, can be more expensive. Daratumumab alone is $120,000 per year and the commonly used VRd regimen is $220,000 for induction treatment. The anticipated D-KRd regimen under investigation is estimated to cost $590,000 [132]. New immunotherapy agents such as Chimeric antigen receptor (CAR) T cells and bispecific T cell engagers (BiTE) are still in development but have shown great promise in myeloma and will certainly become part of the treatment armamentarium and costs. If they follow the same path as B-ALL CAR-T, they will likely cost more than $300,000 and will be used (at least at first) after the other modalities above have failed.

That prices and design of specific regimens may vary can be debated, but the diagnosis of myeloma in the US inarguably brings staggering financial burden. The reasons why cancer drugs are so expensive, whether high prices are justified or not, and potential ways to address these costs have been discussed elsewhere. With the current rate of innovation, we may optimistically hope myeloma will be cured in the next five years, but even if so, significant financial toxicity will persist, and the development of an inexpensive myeloma prevention strategy will remain. Treatments that prevent the development of MM are likely to ease this burden.

## 6. Conclusions

In order to move towards clinical trials in myeloma prevention, we need more research on the basic biology underlying the transition from premalignant to malignant myeloma. The goal of this review is not to change current management at this time, but to raise awareness for the concept of disease prevention in multiple myeloma. Our laboratory is interested in the relationship between obesity, sleep apnea, and myeloma pathophysiology and we feel this deserves closer study. Finally, it is important for healthcare providers to comprehensively evaluate the financial costs of current myeloma treatments at their institutions in order to justify the cost of prevention strategies.

## Figures and Tables

**Figure 1:**
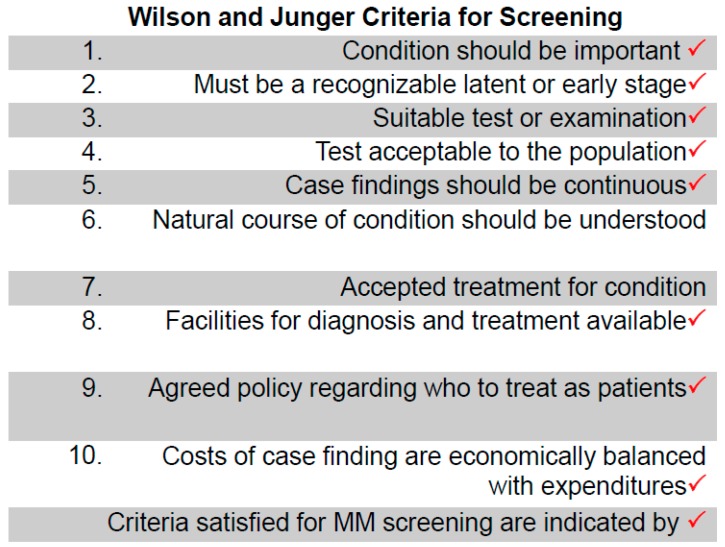
Wilson and Junger criteria for screening. Multiple myeloma meets 8 of 10 criteria necessary for developing a screening and prevention strategy.
